# Seed priming to optimize germination in *Arthrocnemum* Moq.

**DOI:** 10.1186/s12870-022-03893-2

**Published:** 2022-11-14

**Authors:** Esteban Ramírez, Zayneb Chaâbene, Lourdes Hernández-Apaolaza, Mariem Rekik, Amine Elleuch, Vicenta de la Fuente

**Affiliations:** 1 Department of Biology, Universidad Autónoma de Madrid, Cantoblanco, Madrid 28049 Spain; 2 Laboratory of Plant Biotechnology, Faculty of Sciences of Sfax, University of Sfax, Sfax, 3000 Tunisia; 3 Department of Agricultural Chemistry and Food Science, Universidad Autónoma de Madrid, Cantoblanco, Madrid 28049 Spain

**Keywords:** Halophytic crops, Germination, *Arthrocnemum*, Salinity

## Abstract

**Background:**

Seed germination and seedling growth constitute the first stage of a plant's life cycle for crop establishment. *Arthrocnemum* Moq. is a halophyte of the subfamily Salicornioideae (Amaranthaceae), which could be recognized in the foreseeable future as an emerging candidate in applied biosaline agricultural programs, mainly due to the large biomass it represents in coastal and inland saltmarshes, in addition to its interesting nutritional and pharmacological properties. However, to ensure their subsequent use as a crop, it is necessary to optimize their germination through appropriate seed priming treatments. The main goal of this work was to seek the optimization of *Arthrocnemum* germination process using different pretreatments: exposure to sodium chloride (100 to 1200 mM) in the dark and its subsequent transferred to distilled water separately and together with the combination of pH (5, 7, 9), salinity (0, 100, 200 mM NaCl), and iron conditions (0, 200, 400 µM FeSO_4_). The experiments were tested on six samples of two different species: *A*. *meridionale* (from Tunisia) and *A*. *macrostachyum* (from Spain).

**Results:**

Salinity priming of seeds for 15 days in darkness improved germination percentages by almost 25% at 600 mM NaCl, in both Tunisian and Spanish species. However, keeping seeds at different salt concentrations for 30 days produced higher improvement percentages at lower concentrations in *A. meridionale* (100–200 mM NaCl), while in *A. macrostachyum* the highest improvement percentages were obtained at 600 mM NaCl (percentage improvement of 47%). When the dark time period is reduced to 5 days at higher salt concentrations, the greater germination percentages were reached in all the samples at the concentration of 800 mM NaCl, increasing the improvement of germination between 17 and 50%. Finally, the conditions of pH = 7, pretreatment in darkness at 800 mM NaCl and 400 µM or iron, turned out to be an effective medium for seed germination.

**Conclusions:**

Therefore, before using *Arthrocnemum* seeds in applied biotechnological programs, a seed priming treatment based on prior exposure to high salt concentrations (600–1000 mM NaCl) is recommended in order to maximize germination percentages.

**Supplementary Information:**

The online version contains supplementary material available at 10.1186/s12870-022-03893-2.

## Background

Biosaline agriculture involving halophytes is a growing trend due to the ease of application and relatively low cost [1]. Moreover, stressful conditions related to drought, salinity, and high temperatures are gradually promoting the use of salinity-tolerant plant crops. In this regard, halophytes crops are now becoming a more valuable alternative for human nutrition and for other alternative uses [2, 3].

Currently, *Salicornia* L. is the most widely annual halophyte used as a food resource [4–9]. For cultivation, irrigation with 200–400 mM NaCl is recommended for the growth of *Salicornia europaea* L. to achieve optimal properties at the level of biomass and anatomical features of the cultivated plants [10]. However, other perennial halophytes such as *Sarcocornia* A.J. Scott and *Arthrocnemum* Moq. are being evaluated as emergent crops, especially due to their higher biomass and ability to thrive in soils with high salt concentrations [11–14].

*Arthrocnemum* is a genus of halophyte plants belonging to the subfamily Salicornioideae (Amaranthaceae), typical of the Mediterranean region. It grows in saline environments both inland and coastal. Currently, three species are recognized within the genus, *A*. *franzii* Sukhor., exclusively for Cape Verde and some points in Senegal [15, 16]; *A*. *macrostachyum* (Moric.) K. Koch has a distribution by the Western and Eastern Mediterranean subregion and the Alpine-Caucasian subregion; finally, the recently recognized species, *A*. *meridionale* (Ramírez and al.) Fuente and al., can be found on the islands of Sicily and Sardinia and in circum-Mediterranean territories from North Africa (including Tunisia) to the Anatolian Peninsula and the Persian Gulf [17, 18].

Species of the genus *Arthrocnemum* are an emerging halophyte with interesting agricultural and biotechnological uses. [19] demonstrated that *A*. *macrostachyum* is an ideal candidate for extensive Mediterranean green roofs with limited amounts of irrigated water, especially in semiarid and arid coastal areas. Other authors have pointed out several phytochemical properties present in their extracts and tissues for nutrition and human benefit [11, 12, 20, 21]. Consequently, this halophyte is beginning to be used for medicinal crops as well as for the manufacture of biodiesel. According to this last promising application, this succulent shrub has been characterized as one of the most interesting halotolerant species for obtaining bioenergy, based on the quantity and quality of the seed oil [22].

Likewise, it could become a crucial element in saline agriculture for feeding livestock such as camels, sheep, or horses [23–25]. All these potential uses are based on the wide diversity of bioactive compounds found in *Arthrocnemum* plants, mainly rich in phenolic compounds, flavonoids, or fatty acids, among others [14]. Equally important in this plant’s genus is the accumulation of inorganic salts and/or minerals in their tissues [11, 26]. These authors highlighted in samples of *A*. *macrostachyum* collected in Spain (Tinto River, Huelva) and Portugal (Algarve), the high content of inorganic nutrients in its succulent stems, mainly composed of Na, K, Ca, Mg, Fe, Mn, and Zn.

The germination process is the first point that must be optimized for using these types of plants either in the restoration of the vegetation cover or to optimize crop production in agriculture. In addition, the conservation of endangered species or plant endemism depends largely on germination success [27]. However, this process does not always render enough percentage to justify working with these plant species, especially in many taxa of the Amaranthaceae family, in which it is necessary to break the strong dormancy of the seeds through chemical seed priming treatments [28–32].

Seed priming has turned out to be an advantageous method for optimizing crops such as wheat, barley, lentil or cucumber, among others [33]. Halo-priming is one of the most used methods to improve germination in halophytes through inorganic solutions [34, 35]. According to these authors, seed priming begins with the exposure of the seeds to a priming agent (i.e., NaCl, KCl, KNO_3_, CaCl_2_, or plant hormones such as gibberellic acid). Thus, there is an activation of the metabolism of the seed but in which the emergence of the radicle does not occur. Then, the process usually continues with the drying of the seeds after their contact with the priming agent, and finally, the last step consists of rehydrating the seeds in a suitable medium for the successful emergence of the radicle and the subsequent development of the seedlings. The benefits of using priming agents not only promote seed radicle emergence, but also act in synchronizing seed germination and improving the resistance of grown plants against stressful conditions [36].

It is well-documented that other genera evolutionarily related to *Arthrocnemum* such as *Sarcocornia*, *Salicornia,* or *Halocnemum* M. Bieb. (Salicornioideae subfamily), normally have a high percentage of germination without the need for seed priming treatments [37–40]. The facility of germination of these species makes them already suitable for use in applied programs. However, the genus *Arthrocnemum* represent a lineage in which the germination process needs previous treatments to achieve optimal percentages [23, 24, 40–49].

**Fig. 1:**
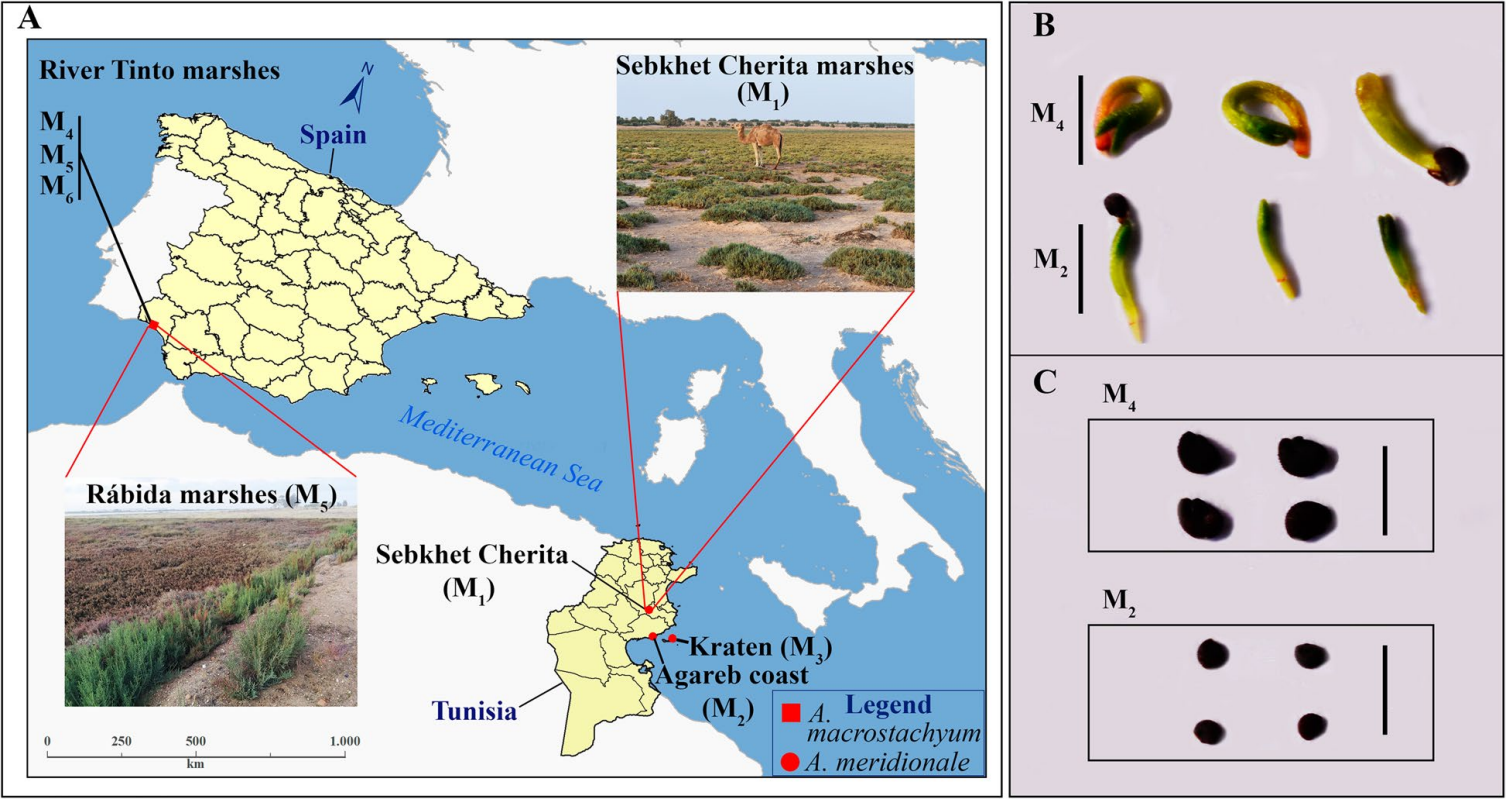
**A** Sampling points and study material. **B** Comparison of germinated seedlings (photographed 19 days after radicle emergence) between *A*. *macrostachyum* (M_4_) and *A*. *meridionale* (M_2_). **C** Comparison of seeds between *A*. *macrostachyum* (M_4_) and *A*. *meridionale* (M_2_). Scale bars 2 mm. Photographs authorship: Esteban Ramírez

In this paper, we focused on *Arthrocnemum* species in the North African Mediterranean (Tunisia) and in the southwestern Mediterranean (Spain) (Fig. 1A), where extensive plant communities are found in inland and coastal saltmarshes and the possibilities of applicational uses have recently been evaluated [50, 51]. Therefore, the aim of this work was to optimize the germination process of the halophyte *Arthrocnemum*, to facilitate its use as an alternative and emerging crop in the Mediterranean area.

## Results

### Generic germination results and seed descriptions

During the experiments, it has been proven that germination started between 2 and 5 days after the seeds were placed in Petri dishes with 0.5% agar (w/v). Between 6 and 19 days the germination process ended completely.

The color of the seeds of both *Arthrocnemum* species varies between black and brown, however, in all samples the black seeds (greater ripeness) were predominant. The mean seed size (length x width) mm was 0.80 × 0.59 in M_1_, 0.77 × 0.6 in M_2_ and 0.72 × 0.56 in M_3_. The mean value of all Tunisian localities was 0.76 × 0.58 mm. The mean values of samples M_4_, M_5_ and M_6_ were 1.22 × 0.92 mm, 1.19 × 0.89 mm, and 1.16 × 0.89 mm, respectively. The average value of the three samples of *A. macrostachyum* is 1.19 × 0.90 mm. Clearly, the Tunisian samples of *A*. *meridionale* species had smaller seeds than *A*. *macrostachyum* species from the plants collected in the Tinto River (Huelva, Spain). Also, the germinated seedlings had less thick radicles and hypocotyls in the Tunisian species compared to the Spanish ones (Fig. 1B and C).

### Control treatment

The percentage of seed germination without prior treatment was relatively low. The results of the control samples in each of the treatments did not exceed 35% of germination in the Tunisian samples M_2_ and M_3_ and it was lower in M_1_ (≤ 15%). In the species *A*. *macrostachyum*, the percentages of germination in the different control treatments were still lower than in *A*. *meridionale*, varying between 9–14% for samples M_4_, M_5_ and M_6_ (Tables 1 and 2).

**Table 1:** Germination percentages of the different pretreatments. M_2_: Agareb coast; M_4_: Tinto River estuary. SD: standard deviation. Values are means ± SD (*n* = 4). d: days (pretreatment duration). The asterisk indicates means within an analyzed variable that is significantly different from the corresponding control treatment (*t*-Student; level of significance (*p* < 0.05))

**Treatment**	**M**_2_	**M**_4_
Germination (%)		
**Control 15d**	32.0 ± 3.3	12.0 ± 3.3
**Control 30d**	35.0 ± 3.8	10.0 ± 2.3
**Darkness + NaCl**		
**15d darkness**		
[NaCl] (mM)		
100	33.0 ± 3.8	13.0 ± 3.8
200	35.0 ± 3.8	15.0 ± 2.0
400	36.0 ± 3.3	15.0 ± 5.0
600	55.0 ± 3.8*	32.0 ± 3.3*
**30d darkness**		
[NaCl] (mM)		
100	63.0 ± 3.8*	16.0 ± 3.3*
200	50.0 ± 5.2*	20.0 ± 3.3*
400	41.0 ± 6.0	23.0 ± 2.0*
600	33.0 ± 5.0	57.0 ± 3.8*

**Table 2:** Germination percentages of the different pretreatments. M_1_: Sebkhet Cherita; M_2_: Agareb coast; M_3_: Kraten; M_4_: Tinto River estuary; M_5_: Rábida; M_6_: San Juan del Puerto. SD: standard deviation. (-) no data. Values are means ± SD (*n* = 4). d: days (pretreatment duration). The asterisk indicates means within an analyzed variable that is significantly different from the corresponding control treatment (*t*-Student; level of significance (*p* < 0.05))

Germination (%)						
**Darkness + NaCl**						
**Treatment**	**M**_**1**_	**M**_**2**_	**M**_**3**_	**M**_**4**_	**M**_**5**_	**M**_**6**_
**Control**	15.0 ± 3.8	29.0 ± 3.8	33.0 ± 3.8	9.0 ± 3.8	12.0 ± 5.7	11.0 ± 2.0
**5d darkness**						
[NaCl] (mM)						
600	15.0 ± 3.8	71.0 ± 7.6*	80.0 ± 9.8*	27.0 ± 7.6*	21.0 ± 7.6	19.0 ± 6.8
800	32.0 ± 8.2*	79.0 ± 6.8*	82.0 ± 6.9*	42.0 ± 5.2*	58.0 ± 5.2*	58.0 ± 7.7*
1000	31.0 ± 8.2*	75.0 ± 11.9*	70.0 ± 8.3*	41.0 ± 7.6*	50.0 ± 2.3*	57.0 ± 8.2*
1200	15.0 ± 5.8	65.0 ± 8.9*	64.0 ± 7.3*	23.0 ± 6.0*	46.0 ± 5.2*	31.0 ± 6.8*
**Combined experiment (pH + NaCl + Fe)**						
**Treatment**	**M**_**1**_	**M**_**2**_	**M**_**3**_	**M**_**4**_	**M**_**5**_	**M**_**6**_
**Control**	13.0 ± 3.8	28.0 ± 3.3	35.0 ± 5.0	9.0 ± 2.0	14.0 ± 4.0	12.0 ± 3.3
**Combined**	40.0 ± 8.6*	78.0 ± 5.2*	83.0 ± 6.8*	39.0 ± 3.8*	60.0 ± 3.3*	54.0 ± 6.9*

### *Darkness* + *salinity pretreatment*

**Fig. 2:**
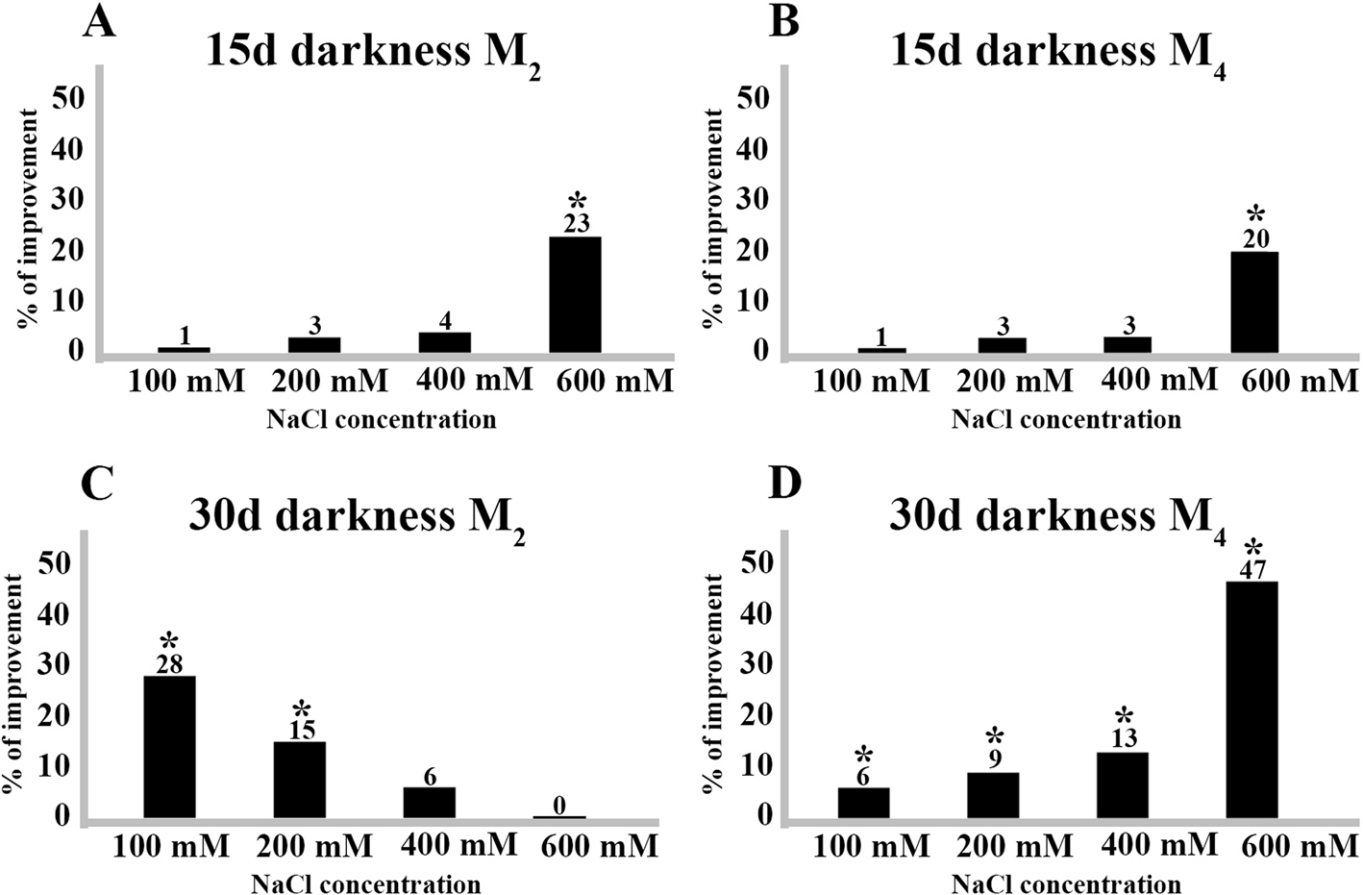
Percentage of improvement obtained by maintaining seeds for 15 and 30 days in darkness at different salinity levels (NaCl) (100, 200, 400 and 600 mM NaCl) with respect to the control (seeds without salt treatment) for the different locations tested: **A**) M_2_ (Agareb coast) and **B**) M_4_ (Tinto River estuary). The asterisk indicates means within an analyzed variable that is significantly different from the corresponding control treatment (*t*-Student; level of significance (*p* < 0.05))

After 15 days of keeping seeds from M_2_ and M_4_ in the dark at the saline concentrations of 100, 200, 400 and 600 mM NaCl, the following results were obtained: at the concentrations of 100, 200 and 400 mM the germination percentages were not statistically different from the control. However, a clear result of this experiment with statistical support was obtained with 600 mM, increasing the germination percentage by 23% and 20% compared to the control treatment in each of the respective samples (Table 1, Fig. 2A and B).

After 30 days in these conditions the results were different between species. In *A*. *meridionale* (M_2_) the best germination percentages with a mean of 63% were obtained at 100 mM NaCl. Increasing salt concentration for 30 days, decreased seeds germination percentage gradually and with 600 mM NaCl, the untreated control was more effective than the pretreatment used (Table 1, Fig. 2C). However, different results have been shown for *A*. *macrostachyum* species (M_4_). Significant differences were observed between the control treatment and the priming seed treatment at different salinity concentrations with 100, 200, 400 and 600 mM NaCl. At the concentration of 600 mM NaCl a high improvement of 47% was achieved for this plant sample, which implies average germination percentages of 57% (Table 1, Fig. 2D).

**Fig. 3:**
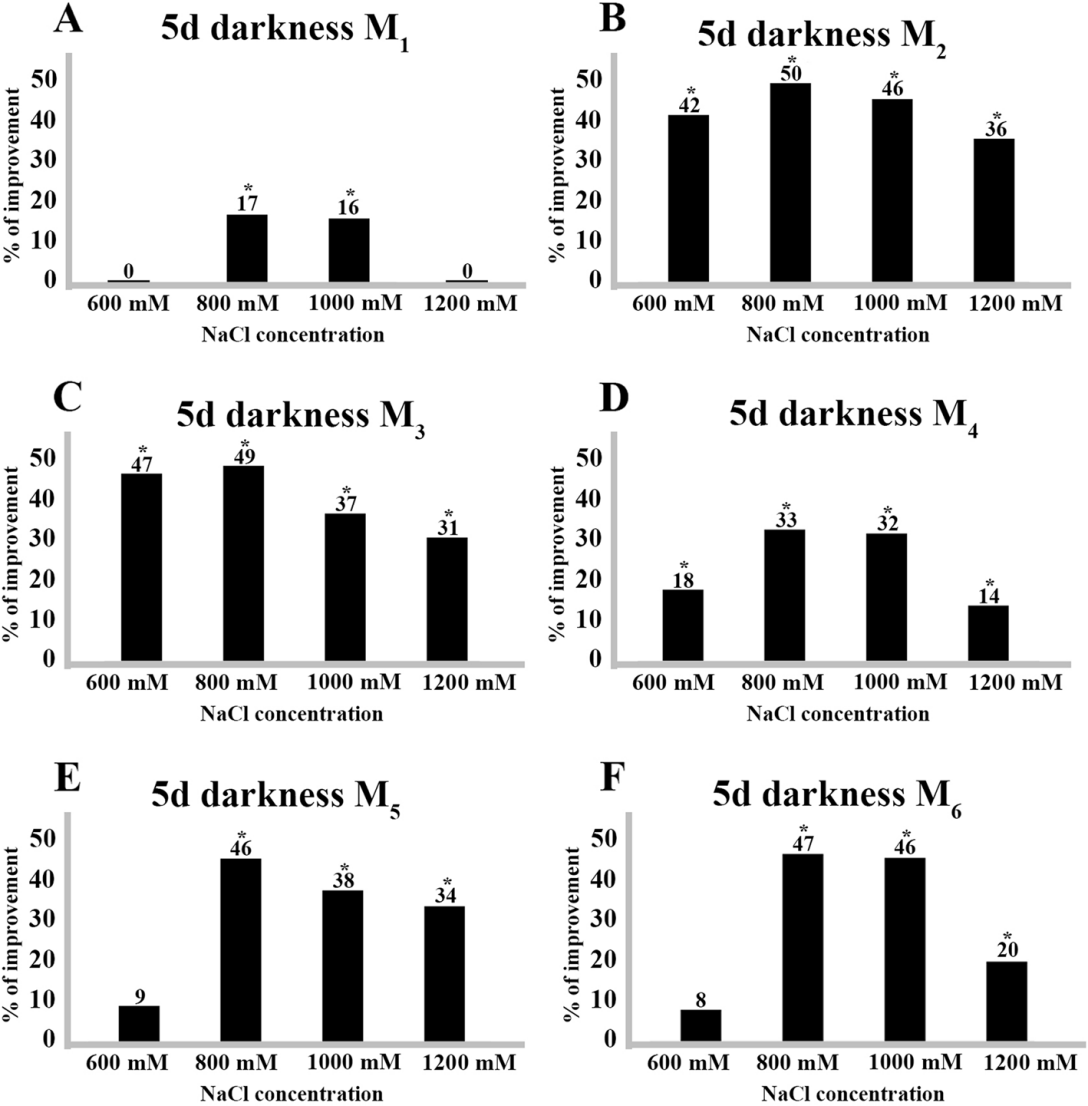
Percentage of improvement obtained by maintaining seeds 5 days in darkness at different salinity levels (NaCl) (600, 800, 1000 and 1200 mM NaCl) with respect to the control (seeds without treatment) for the different locations tested: **A**) M_1_ (Sebkhet Cherita), **B**) M_2_ (Agareb coast), **C**) M_3_ (Kraten), **D**) M_4_ (Tinto River estuary), **E**) M_5_ (Rábida) and **F**) M_6_ (San Juan del Puerto). The asterisk indicates means within an analyzed variable that is significantly different from the corresponding control treatment (*t*-Student; level of significance (*p* < 0.05))

Increasing the salt concentrations to 600, 800, 1000 and 1200 mM NaCl and decreasing the exposure to darkness time to 5 days in all samples, the percentages of seeds germination were improved compared to the control treatment with strong statistical support, except for M_1_ samples (at 600 mM and 1200 mM NaCl) and M_5_ and M_6_ (at 600 Mm NaCl). The best percentages of improvement occurred at a concentration of 800 mM NaCl in the six samples tested (Table 2, Fig. 3).

The M_1_ sample of *A. meridionale* showed lower improvement percentages than those of the M_2_ and M_3_ samples of the same species, with only maximum final germination percentages of 32% at 800 mM NaCl (Table 2, Fig. 3A). For these latest Tunisian samples M_2_ and M_3_, the best germination results ranging from 70 to 82% were obtained for the concentrations of 600, 800 and 1000 mM NaCl. This percentage of improvement was slightly decreased at the concentration of 1200 mM NaCl for both M_2_ and M_3_ samples. Although there was a slight decrease in these ranges, this yield was generally high as the average percentage of germinated seeds in these two Tunisian samples at all concentrations varied between 64 and 82% (Table 2, Fig. 3B and C). The three samples of the species *A. macrostachyum* (M_4_, M_5_ and M_6_) had a highly similar germination pattern: at 600 and 1200 mM NaCl the improvement percentage was lower (8%—34%) than at concentrations of 800 and 1000 mM NaCl (32%-47%).

The germination percentages in samples M_5_ and M_6_ were slightly higher than those obtained in M_4_, except for the concentration of 600 mM NaCl. This sample from the Tinto River estuary (M_4_) reached, at a concentration of 600 mM, a mean germination percentage of 27% whereas in M_5_ and M_6_ it was 21% and 19%, respectively (Table 2, Fig. 3D, E and F).

### pH, salinity, and iron experiment

The best germination conditions with statistical support (Duncan test; *p *< 0.05) came from experiment T8 (pH: 7; salinity: 0; iron: 400 µM), shown in Table 3. In this treatment, the average germination percentage was 15% in M_1_, 56% in M_2_ and 59% in M_3_. For the rest of the experiments there were differences between samples, however, the conditions of pH, salinity and iron established by treatments T11 for M_1_, T7 for M_2_ and T6 for M_3_, consecutively followed treatment T8 as the next most effective. In *A. macrostachyum* samples (M_4_-M_6_) the final germination percentages were very low and did not allow any conclusions to be drawn (data not shown).

**Table 3:** Germination percentage of the pH, salinity, and iron experiment with 15 different combined treatments for three different factors following a Box-Behnken Design (BBD). M_1_: Sebkhet Cherita; M_2_: Agareb coast; M_3_: Kraten. Data are given as Mean (*n* = 4) ± SD (standard deviation). Column M_1_, M_2_, M_3_, one-way ANOVA. Different letters within an analyzed column (M_1_, M_2_ and M_3_ individually each one) indicate significant differences in the germination means for the 15 treatments; the further away each letter is from letter a means the higher the germination percentage in the specific treatment (Duncan test; significance of (*p* < 0.05))

**Germination (%)**			
**Treatments**	**M**_**1**_	**M**_**2**_	**M**_**3**_
**T1**	8.0 ± 3.3^**abc**^	36.0 ± 5.7^** cd**^	47.0 ± 8.2^** fg**^
**T2**	4.0 ± 3.3^**ab**^	19.0 ± 6.8**ª**	33.0 ± 11.5^**bcd**^
**T3**	6.0 ± 5.2^**abc**^	39.0 ± 6.8^**de**^	44.0 ± 4.6^**defg**^
**T4**	2.0 ± 2.3^**a**^	34.0 ± 9.5^** cd**^	19.0 ± 8.2^**a**^
**T5**	9.0 ± 2.0^**bcd**^	28.0 ± 5.7^**abc**^	35.0 ± 6.0^**bcdef**^
**T6**	9.0 ± 3.8^**bcd**^	35.0 ± 5.0^** cd**^	52.0 ± 5.7^**gh**^
**T7**	6.0 ± 4.0^**abc**^	48.0 ± 5.7^**ef**^	45.0 ± 6.0^**defg**^
**T8**	15.0 ± 6.0^**d**^	56.0 ± 4.6^**f**^	59.0 ± 2.0^** h**^
**T9**	7.0 ± 3.8^**abc**^	40.0 ± 6.5^**de**^	34.0 ± 4.0^**bcde**^
**T10**	4.0 ± 3.3^**ab**^	34.0 ± 5.2^** cd**^	39.0 ± 2.0^**bcdef**^
**T11**	8.5 ± 3.4^**abc**^	47.0 ± 5.0^**ef**^	46.0 ± 7.7^**efg**^
**T12**	9.0 ± 3.8^**bcd**^	22.0 ± 4.0^**ab**^	27.0 ± 5.0^**ab**^
**T13**	5.0 ± 3.8^**abc**^	27.0 ± 9.5^**abc**^	30.0 ± 9.5^**abc**^
**T14**	2.0 ± 2.3^**a**^	27.0 ± 8.2^**abc**^	28.0 ± 14.6^**ab**^
**T15**	11.0 ± 6.8^** cd**^	31.0 ± 6.0^**bcd**^	41.0 ± 5.0^**cdefg**^

### Combined experiment

**Fig. 4:**
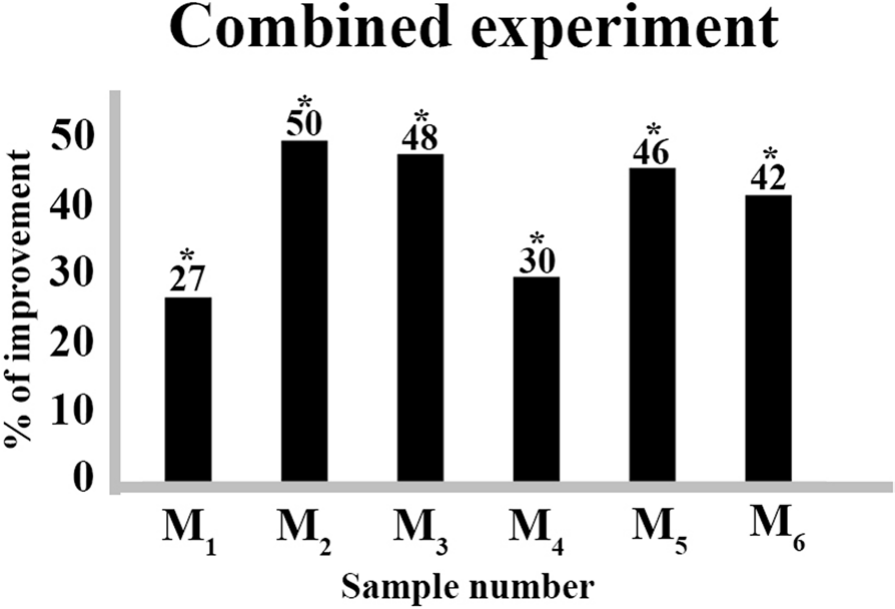
Percentage of improvement obtained by the best combination obtained in the pH, salinity, and iron experiment (treatment T8), in combination with seed pretreatment for five days in darkness immersed in 800 mM saline solutions of NaCl, in comparison to the untreated control for the different locations tested: M_1_ (Sebkhet Cherita), M_2_ (Agareb coast), M_3_ (Kraten), M_4_ (Tinto River estuary), M_5_ (Rábida) and M_6_ (San Juan del Puerto). The asterisk indicates means within an analyzed variable that is significantly different from the corresponding control treatment (*t*-Student; level of significance (*p* < 0.05))

Optimal pH, salinity, and iron germination protocol (treatment T8), together with the combined effect of darkness (5d) + salinity (800 mM of NaCl) pretreatments produced the following results: regarding all previous experiments, it reached the highest germination percentage for the M_1_ sample (40%), with an improvement in germination compared to its control of 27% (Table 2 and Fig. 4).

High germination percentages with statistical support were obtained in samples M_2_ and M_3_, 78% and 83% respectively (Table 2 and Fig. 4). Germination in samples M_4_, M_5_ and M_6_ was also improved following this protocol (mean final germination ranged from 39–60%). (Table 2 and Fig. 4).

## Discussion

### First steps in the germination of complex taxonomic groups

Nomenclatural and taxonomic clarifications allow comparisons of germination results with greater evolutionary affinity. It is of great importance to take this fact into account within the Salicornioideae subfamily, as it is considered one of the most conflictive halophyte groups. In this subfamily of Amarathaceae, the seed characters based on color, shape, size, macro and microornamentation characteristics allow us to classify these halophytes at the level of genus, species and even in certain groups of subspecies [17, 18, 52–55].

In this article we include comparative images, specific collection sites and seed sizes (length and width) of the species *A. meridionale* and *A. macrostachyum*. This detailed description of the characteristics of the seeds and the geographical environment of the analyzed samples will serve for future compendia on the germination capacity of both species and, ultimately, our data may increase the chances of success in applied saline agricultural programs. For instance, *Arthrocnemum indicum* (Willd.) Moq, nomenclatural synonym of *Tecticornia indica* (Willd.) K.A.Sheph. and Paul G.Wilson, is sometimes used as a species that really belongs to the genus *Arthrocnemum* (Sebkha of Sidi el Heni, southwest of Sousse, Tunisia) [51], since *T. indica* is absent in Tunisia [15]. While other times the name *A. indicum* is used to actually refer to its taxonomic synonym *T. indica* [47, 49]. This fact greatly hinders the unification of germination results referred to the genus *Arthrocnemum sensu stricto*. In this regard, we highlight the need to include in germination studies a detailed description of the species to be treated, especially important in groups of plants with complex taxonomy.

### Seed priming treatment on *Arthrocnemum* germination

In traditional studies on tolerance of seeds to salinity without enforced dormancy pretreatments under these stress conditions, it has been shown that in *Arthrocnemum*, germination is reduced due to an increase in salinity and, normally, the untreated control produced higher germination percentages [23, 27, 40, 43, 44, 46, 48, 49, 56]. The first germination test on *Arthrocnemum* found in the literature was performed by [56] from the “Le cap Croisette” (southern Marseille, France). The author exposed twenty seeds per treatment to different progressive concentrations of the chlorine ion (0 to 45 g/l) and the main conclusions in these experiments were that the number of germinations decreased with increasing chlorinity until the germination process was suppressed, with Cl^−^ concentrations of 40 g/l, corresponding approximately to twice sea ​​water chlorinity.

Seed dormancy has been described as the incapacity of a viable seed to germinate under favorable conditions [57, 58]. As a result, the low germination percentages tested on plants from dormant seeds make them unsuitable for use in agriculture or in a variety of other alternative applications. Seed priming constitutes an important biotechnological tool to promote dormant seed germination in addition to producing greater seedling resistance to salinity stress conditions. While it is true that the improvement of germination through seed priming treatments depends among other factors on the type of priming agent, the duration of exposure of the seeds to the agent, and its concentration [34, 35, 59, 60]. Seed priming phases begin with their imbibition in a priming agent, followed by dehydration of the seeds and finally a re-imbibition in water or in a medium favorable for germination [61]. In our experiments the dehydration period, after exposure for several days to different salt concentrations and after subsequent washing in distilled water, was reduced to a minimum (5 min under Bunsen flame). Then by rehydrating the seeds in 0.5% agar medium our results showed higher germination percentages than the control treatment in most trials.

An adequate medium for the germination of *Arthrocnemum* seeds was the one obtained in the experiment T8 of pH, salinity, and iron (where pH: 7; salinity: 0 mM and iron: 400 µM). In the combined experiment (included pH control, pretreatment at high salt concentration and subsequent transfer to a medium without salinity, and a certain amount of iron), it produced higher percentages of improved germination with respect to the control treatments. In this sense, [62] tested the effect of iron toxicity on *Vigna radiata*. From 300 µM of iron, the germination percentage decreased drastically with respect to the control, while with 500 µM germination was paralyzed. *Arthrocnemum* seeds have been found to be more tolerant to iron than *V. radiata* seeds. In fact, seed germination in *Arthrocnemum* was favored at 400 µM FeSO_4_.

Another type of previous treatment to promote germination has been based on its exposure to a certain period of cold. [48] found that without a seed priming treatment the germination percentage in *Arthrocnemum* plants collected from northern Algeria was quite low, ranging from 5 to 30% depending on the germination temperature (30% to 40ºC). To achieve an improvement in germination, they exposed the seeds to a cold pretreatment without salinity regime (5ºC for 2, 10 and 27 weeks) and the germination percentages increased 30%, 40% and 80% respectively. However, the pretreatment time necessary to achieve a high percentage of germination was very long, which makes it difficult to implement it as a useful tool in the applied programs. Seed priming through different concentrations of sodium chloride rapidly improve germination percentages in *Arthrocnemum*. In the experiments carried out on *A*. *macrostachyum* and *A*. *meridionale*, the pretreatment with NaCl and its subsequent transferred to distilled water improved germination by breaking the strong dormancy of the seeds. Pretreatment in the dark and salinity for 15 days showed that for both species the concentration of 600 mM NaCl produced a greater improvement in germination. At 30 days there were differences between plants populations: *A. meridionale* had better results at the concentration of 100 mM NaCl (28% improvement compared to the control) while *A*. *macrostachyum* at 600 mM NaCl (47% improvement). Exposure for 30 days with high concentrations of NaCl negatively affected germination in *A. meridionale* while for *A*. *macrostachyum* the process improved. It seems that the different degree of dormancy and the collection date could play a key role (August in *A*. *macrostachyum* and October in *A*. *meridionale*). In fact, within *A. meridionale* group, M_1_ sample collected in September had less germination success than the M_2_ and M_3_ samples collected in October of the same year. On the contrary, the samples M_4_, M_5_ and M_6_ of *A. macrostachyum* were collected in the same week of the same month (August) and the germination results were more homogeneous between samples. It could be very interesting in future studies to verify, based on a high sample number and during several years of sampling, whether or not the month of collection of the seeds have or not a significant influence on the final percentage of germination in *Arthrocnemum*.

It is well known that pretreatment with high salt concentrations and subsequent relief in a non-saline medium improved the germination process in halophytes [63–65]. In our samples, exposure for 5 days to 600, 800, 1000 and 1200 mM NaCl and its subsequent transferred to a distilled water generated better results when the concentrations of 800 and 1000 were used. However, frequently the concentration of 600 mM NaCl produced similar results to those obtained from 800 and 1000 mM NaCl (sample M_3_). In accordance with our data, [43] mainly demonstrated that in *A. macrostachyum* when saline stress is relieved with higher osmotic potentials, the success of germination increased in the four salts used (MgCl_2_, MgSO_4_, NaCl and Na_2_SO_4_) as pretreatments. The maximum germination percentage reached in this species from low (3.90 MPa) to high (0.00 MPa) osmotic potential pretreatment was approximately 65%. [23] found that the *Arthrocnemum* seeds pretreated for 20 days with 600, 800 and 1000 mM NaCl salt and subsequently transferred to distilled water had higher germination rates compared to the control samples. [44] also found in this halophyte genus that the seeds pre-treated for 30 days with 5% and 10% NaCl, showed germination percentages of 44% and 62% respectively, compared to the control test which was 26%. This implied improvement percentages of 18% at a concentration of 5% NaCl and 36% at a concentration of 10%.

Along the same lines, [49] subjected *Arthrocnemum* seeds to different saline solutions. They verified that the seeds could not germinate at high NaCl concentrations, however, when subsequently transferred to plates with only distilled water, a dormancy break occurred, and the germination percentage became higher than in the control treatments. Like us, all of these authors proved that salt pretreatment broke seed dormancy in *Arthrocnemum*. The ability of the seeds of many halophytes to remain inactive at high saline concentrations in the soil and its germination during rainfall periods and lower salinity gives this group a strong evolutionary advantage over glycophytes [66].

### Future applications after the improvement of the germination process in *Arthrocnemum*

*Arthrocnemum* plants from Tunisia have been nutritionally evaluated and recognized as a halophyte that could be considered as a healthy food due to the high levels of dietary fibers, protein, and polyunsaturated fatty acids (especially relevant in linolenic acid), in its shoots in two seasonal/phenotypic stages (green shoots and red-violet shoots). Likewise, it was considered as an important source of minerals, particularly sodium, calcium, potassium, magnesium as well as vitamins C and E [51]. In Spain it has also been very recently revalued based on the detection of bioactive compounds. [14] found phenolic acids (salicylic acid, veratric acid, caffeic acid, coumaric acid, transcinnamic acid and ferulic acid); flavonoids (p-Coumaroyl-glucoside, dihydroquercetin and luteolin) and numerous fatty acids, including palmitic acid, oleic acid, stearic acid, linoleic acid, linolenic acid, behenic acid and lignoceric acid, among others. This great variety of bioactive compounds detected confers an additional nutritional and pharmacological value to the species *A. macrostachyum* due to its antioxidant properties and its contribution of essential fatty acids for the human diet and for livestock. In addition, it has been observed that the content of total polyphenols in *A. macrostachyum* increases significantly when treated at moderate and high salinity levels (200 and 400 mM NaCl), which would improve the nutritional properties of *Arthrocnemum* crops through irrigation with saline water [67].

Furthermore, this perennial succulent shrub could have great potential in the restoration of the vegetation cover (greater or lesser tolerance to salinity could have an important effect on the dominance of plant communities with respect to other plants whose germination is inhibited by high concentrations of salt in the soil), as well as in the reduction of salinity in soils that could later be used for agricultural purposes [49, 50]. Furthermore, *Arthrocnemum* could be used in intercropping systems with other non-halophilic crops of commercial interest, since it could help mitigate the salt stress for sensitive crops, as occurs in the mixed crop of tomato (*Lycopersicon esculentum* Mill) with various halophytes [68].

Also, it was also found that the seeds subjected to priming treatments, apart from improving their germination, the subsequent seedlings turned out to be more tolerant and resistant to saline stress conditions [58, 69]. Therefore, the optimization of the germination process through adequate seed priming treatment allows the availability of stable and abundant seedlings of *Arthrocnemum* plants for their future use in saline agriculture, ecosystem restoration or other biotechnological uses.

## Conclusions

Germination in the genus *Arthrocnemum* has been highly favored based on seed priming treatment. Previous exposure of seeds to high concentrations of salt (between 600–1200 mM NaCl for 5–15 days depending on seed dormancy), and their subsequent transfer to distilled water or a favorable medium (pH: 7; salinity: 0; iron: 400 µM) showed a significant improvement in the germination of the two species *A. meridionale* and *A. macrostachyum*. The most effective concentration in most of the improvement treatments carried out in this study corresponded to 800 mM NaCl, reaching improvement percentages of up to 50% compared to the corresponding control treatment. For future research that focuses on this genus we recommend promoting a break in dormancy through seed priming treatments before using *Arthrocnemum* seeds in applied biosaline agriculture programs or other alternative uses. The abundance of these species in Tunisia, as well as in Spain and in other Mediterranean countries, make *Arthrocnemum* a prime candidate on which to focus studies, especially since this halophyte is gradually becoming an emerging edible plant with recognized nutritional and pharmacological properties.

## Methods

### Study species and plant identification

Inflorescences of *A*. *meridionale* (M_1_-M_3_) and *A. macrostachyum* (M_4_-M_6_) plants were collected during August-October 2019 at three different locations in Tunisia and Spain: M_1_. Sebkhet Cherita (Mahdia, Tunisia), 32SPE1309, 23–09-2019, leg. E. Ramírez, E. and A. Khardani, MAF 178644. M_2_. Agareb coast near to Gargour (Sfax, Tunisia), 32SPD5333, 12–10-2019, leg. E. Ramírez and M. Rekik, MAF 180597. M_3_. Kraten saltmarsh (Chergui, Kerkennah Archipelago, Sfax, Tunisia), 32SQD0554, 17–10-2019, leg. E. Ramírez and M. Rekik, MAF 180599. M_4_. Tinto River estuary (Huelva province, Spain), 29SPB8220, 05–08-2019, leg. E. Ramírez, MAF 180596. M_5_. Rábida marshes, Tinto River (Huelva province, Spain), 29SPB8420, 06–08-2019, leg. E. Ramírez, MAF 180598. M_6_. San Juan del Puerto marshes, Tinto River (Huelva province, Spain), 29SPB9131, 07–08-2019, leg. E. Ramírez, MAF 180595. The first sampling point, Sebkhet Cherita represents an inland saline area, the second Agareb coast collection point has the influence of the sea and the third point, Kraten saltmarsh, is a small island of an archipelago also influenced by the proximity of the sea. The Spanish localities collected from the Tinto River saltmarshes, an environment highly rich in heavy metals such as iron, comprise three points that include, first of all, the mouth of the Tinto River together with the Odiel River in the Atlantic Ocean (Tinto River estuary); then an intermediate saltmarsh area (Rábida) and finally, a sampling point that constitutes the northernmost limit of the saltmarsh formations in the Tinto River (San Juan del Puerto). The study area is exposed in Fig. 1A. Photographs of 20 seeds with the Olympus SC30 camera integrated into a stereomicroscope (Olympus SZX10) were taken for each sample. From these data, measurements of their length–width were made with the Image J program (Java 1.8.0). The measurements were compared with the ranges established in [17, 18]. The plant material was deposited in the MAF herbarium (Faculty of Pharmacy Herbarium, Complutense University of Madrid).

### General protocol

Ripe black seeds were manually separated from fruits and stored in dry papers at room temperature. The dehydrated seeds were stored from the date of collection of each of the samples until the beginning of the laboratory experiments in November–December 2019. Then the surface was sterilized with different concentrations of sodium hypochlorite solutions (2 min at 50%, 10 min at 20% and 30 min at 10%) and washed three times with autoclave distilled water before germination. Germination was carried out in Petri dishes (9 cm) with 0.5% (w/v) agar which were placed in a growth chamber maintained at 24 °C ± 2 °C with a 16/8 h photoperiod for 21 days. Percentage of germination was recorded every 2–3 days. Seeds were considered to be germinated after the radicle emergence. A full experiment was performed under sterile conditions and Petri dishes were sealed with parafilm paper to prevent their opening and contamination in the growth chamber. For each of the treatments, 4 replicates with 25 seeds per Petri dish were carried out.

Percentage of individual germination for each treatment, and a percentage of improvement with respect to the control treatment were determined as:

- Final germination percentage (x̄): (a/b) *100; where a is the number of seeds germinated and b is the total number of seeds tested.
- Average percentage: (x̄_R1_ + x̄ _R2_ + x̄ _R3_ + x̄ _R4_)/4; where x̄_R_ is each of the four replicates.
- Improvement (%) = (average percentage of pretreatment) – (average percentage of control treatment).

### Darkness salinity pretreatment

Seeds from a Tunisian locality (M_2_) and from Spain (M_4_) were kept for either 15 days or 30 days in darkness at different salinity concentrations of 0 (control treatment), 100, 200, 400 and 600 mM NaCl. The experiment was repeated with concentrations of 0 (control treatment), 600, 800, 1000 and 1200 mM for the six samples and were kept for 5 days in darkness. Then, the seeds were washed three times with autoclaved distilled water, dried for five minutes under Bunsen flame and then transferred to Petri dishes with 0.5% agar (w/v), following the general protocol.

### pH, salinity, and iron pretreatment

The experiment was carried out using three factors (pH, salinity, and iron) and three levels of each factor: pH (5, 7 and 9); salinity (0, 100 and 200 mM NaCl) and iron (0, 200 and 400 µM FeSO_4_). Method used by [70] was followed to perform the Box-Behnken Design (BBD) in order to achieve high variability in experiments of fifteen treatments for three different factors. In the protocol, 50 ml solutions were prepared, to which, depending on the experiment number (treatment T1-T15), different amounts of sodium chloride (0, 100 mM or 200 mM) and iron (II) sulfate (0, 200 and 400 µM), were added. A 0.5% (w/v) concentration of agar was also included to each of the solutions. These products were mixed and subsequently the pH was adjusted to 5, 7 or 9 for each of the solutions. The different mixtures were sterilized in an autoclave and transferred to Petri dishes, following the general protocol. The central values (zero level) chosen for the experimental design were as follows: pH (7), salinity (100 mM NaCl) and iron (200 µM). The combinations used in the experiments are shown in Table 4.

**Table 4:** Different pH (5, 7, 9), salinity (NaCl 0, 100 mM, 200 mM) and iron (FeSO_4_, 0, 200 µM, 400 µM) treatments used at the experiment following a Box-Behnken Design (BBD)

**Treatment**	**T1**	**T2**	**T3**	**T4**	**T5**	**T6**	**T7**	**T8**	**T9**	**T10**	**T11**	**T12**	**T13**	**T14**	**T15**
**pH**	5	7	7	5	7	7	5	7	7	7	9	9	9	5	9
**Salinity (mM)**	0	0	100	100	200	100	100	0	100	200	100	0	100	200	200
**Iron (µM)**	200	0	200	0	0	200	400	400	200	400	0	200	400	200	200

### Combined experiment

The best combination obtained in the pH, salinity, and iron experiment, with statistical approval, was used to seek further optimization of the germination process in combination with seed pretreatment for five days in darkness immersed in 800 mM saline solutions of NaCl. Then seeds were washed by three times with autoclaved distilled water and transferred to Petri dishes with 0.5% agar (w/v) and the best pH, salinity, and iron conditions.

### Statistical analysis

The mean germination percentage and its standard deviations were calculated for each of the experiments with IBM SPSS Statistics 26 software. Differences between control and specific pretreatment for each of the samples were compared by the *t*-Student test (level of significance *p* < 0.05) (Table 1). For the pH, salinity and iron experiment, a one-way ANOVA was performed to compare each individual sample (M_1_, M_2_ and M_3_) with each of the 15 treatments. The cases responsible for significant main effects were detected using the Duncan test (level of significance *p* < 0.05).

## Supplementary Information

**Additional file 1.**

## Data Availability

All data generated or analysed during this study are included in this published article [and its supplementary information files].
