# Supplementary material for: Seed priming to optimize germination in Arthrocnemum Moq
Source: BMC Plant Biol. 2022 Nov 14;22:527. doi: 10.1186/s12870-022-03893-2 (PMC9661790; doi:10.1186/s12870-022-03893-2)
Supplement: Supplementary file 1 — Additional file 1. [file 12870_2022_3893_MOESM1_ESM.docx]

| TREATMENT | Control M2 | Control M4 | 15dM2_100 | 15dM2_200 | 15dM2_400 | 15dM2_600 | 15dM4_100 | 15dM4_200 | 15dM4_400 | 15dM4_600 |
| --- | --- | --- | --- | --- | --- | --- | --- | --- | --- | --- |
| R1 | 28 | 8 | 36 | 32 | 36 | 52 | 12 | 16 | 16 | 32 |
| R2 | 32 | 12 | 28 | 36 | 32 | 56 | 16 | 12 | 8 | 28 |
| R3 | 36 | 12 | 32 | 40 | 40 | 52 | 16 | 16 | 16 | 32 |
| R4 | 32 | 16 | 36 | 32 | 36 | 60 | 8 | 16 | 20 | 36 |
| MEAN | 32,0 | 12,0 | 33,0 | 35,0 | 36,0 | 55,0 | 13,0 | 15,0 | 15,0 | 32,0 |
| SD | 3,27 | 3,27 | 3,83 | 3,83 | 3,27 | 3,83 | 3,83 | 2,00 | 5,03 | 3,27 |
| Improvement | - | - | 1,0 | 3,0 | 4,0 | 23,0 | 1,0 | 3,0 | 3,0 | 20,0 |

**SUPPLEMENTARY INFORMATION FILES**

**Table S1.** Germination percentages of the darkness salinity pretreatment (15 days). M2: Agareb coast; M4: Tinto River estuary. R1, R2, R3 and R4 (four replicates of 25 seeds each one). SD: standard deviation. Raw data.

**Table S2.** Germination percentages of the darkness salinity pretreatment (30 days). M2: Agareb coast; M4: Tinto River estuary. R1, R2, R3 and R4 (four replicates of 25 seeds each one). SD: standard deviation. Raw data.

| TREATMENT | Control M2 | Control M4 | 30dM2_100 | 30dM2_200 | 30dM2_400 | 30dM2_600 | 30dM4_100 | 30dM4_200 | 30dM4_400 | 30dM4_600 |
| --- | --- | --- | --- | --- | --- | --- | --- | --- | --- | --- |
| R1 | 40 | 12 | 60 | 52 | 36 | 32 | 20 | 16 | 24 | 60 |
| R2 | 32 | 8 | 68 | 56 | 48 | 32 | 12 | 24 | 24 | 52 |
| R3 | 32 | 12 | 64 | 48 | 44 | 40 | 16 | 20 | 20 | 56 |
| R4 | 36 | 8 | 60 | 44 | 36 | 28 | 16 | 20 | 24 | 60 |
| MEAN | 35,0 | 10,0 | 63,0 | 50,0 | 41,0 | 33,0 | 16,0 | 20,0 | 23,0 | 57,0 |
| SD | 3,83 | 2,31 | 3,83 | 5,16 | 6,00 | 5,03 | 3,27 | 3,27 | 2,00 | 3,83 |
| Improvement | - | - | 28,0 | 15,0 | 6,0 | -2,0 | 6,0 | 10,0 | 13,0 | 47,0 |

**Table S3.** Germination percentages of the darkness salinity pretreatment (5 days). M1: Sebkhet Cherita; M2: Agareb coast; M3: Kraten; M4: Tinto River estuary; M5: Rábida; M6: San Juan del Puerto. R1, R2, R3 and R4 (four replicates of 25 seeds each one). SD: standard deviation. Raw data.

| TREATMENT | Control M1_5d | Control M2_5d | Control M3_5d | Control M4_5d | Control M5_5d | Control M6_5d |
| --- | --- | --- | --- | --- | --- | --- |
| R1 | 12 | 32 | 28 | 4 | 20 | 12 |
| R2 | 20 | 28 | 36 | 12 | 8 | 12 |
| R3 | 12 | 24 | 36 | 12 | 8 | 12 |
| R4 | 16 | 32 | 32 | 8 | 12 | 8 |
| MEAN | 15,0 | 29,0 | 33,0 | 9,0 | 12,0 | 11,0 |
| SD | 3,8 | 3,8 | 3,8 | 3,8 | 5,7 | 2,0 |
| Improvement | - | - | - | - | - | - |

| 5dM1_600 | 5dM1_800 | 5dM1_1000 | 5dM1_1200 | 5dM2_600 | 5dM2_800 | 5dM2_1000 | 5dM2_1200 | 5dM3_600 | 5dM3_800 | 5dM3_1000 | 5dM3_1200 | 5dM4_600 | 5dM4_800 | 5dM4_1000 | 5dM4_1200 | 5dM5_600 |
| --- | --- | --- | --- | --- | --- | --- | --- | --- | --- | --- | --- | --- | --- | --- | --- | --- |
| 12 | 28 | 20 | 20 | 76 | 76 | 88 | 68 | 88 | 72 | 68 | 56 | 32 | 44 | 52 | 20 | 16 |
| 20 | 40 | 32 | 12 | 60 | 88 | 80 | 60 | 68 | 88 | 60 | 68 | 16 | 40 | 36 | 16 | 16 |
| 12 | 32 | 40 | 16 | 72 | 72 | 72 | 76 | 88 | 84 | 72 | 60 | 28 | 36 | 36 | 28 | 20 |
| 16 | 28 | 32 | 12 | 76 | 80 | 60 | 56 | 76 | 84 | 80 | 72 | 32 | 48 | 40 | 28 | 32 |
| 15,0 | 32,0 | 31,0 | 15,0 | 71,0 | 79,0 | 75,0 | 65,0 | 80,0 | 82,0 | 70,0 | 64,0 | 27,0 | 42,0 | 41,0 | 23,0 | 21,0 |
| 3,8 | 5,7 | 8,2 | 3,8 | 7,6 | 6,8 | 11,9 | 8,9 | 9,8 | 6,9 | 8,3 | 7,3 | 7,6 | 5,2 | 7,6 | 6,0 | 7,6 |
| 0,0 | 17,0 | 16,0 | 0,0 | 42,0 | 50,0 | 46,0 | 36,0 | 47,0 | 49,0 | 37,0 | 31,0 | 18,0 | 33,0 | 32,0 | 14,0 | 9,0 |

**Table S4.** Germination percentages of the darkness salinity pretreatment (combined experiment). M1: Sebkhet Cherita; M2: Agareb coast; M3: Kraten; M4: Tinto River estuary; M5: Rábida; M6: San Juan del Puerto. R1, R2, R3 and R4 (four replicates of 25 seeds each one). SD: standard deviation. Raw data.

| TREATMENT | Control M1_5d | Control M2_5d | Control M3_5d | Control M4_5d | Control M5_5d | Control M6_5d | CM1 | CM2 | CM3 | CM4 | CM5 | CM6 |
| --- | --- | --- | --- | --- | --- | --- | --- | --- | --- | --- | --- | --- |
| R1 | 16 | 28 | 36 | 8 | 16 | 16 | 52 | 72 | 80 | 44 | 60 | 56 |
| R2 | 12 | 32 | 28 | 12 | 16 | 12 | 36 | 76 | 76 | 36 | 60 | 56 |
| R3 | 8 | 24 | 36 | 8 | 8 | 8 | 40 | 84 | 84 | 40 | 56 | 44 |
| R4 | 16 | 28 | 40 | 8 | 16 | 12 | 32 | 80 | 92 | 36 | 64 | 60 |
| MEAN | 13,0 | 28,0 | 35,0 | 9,0 | 14,0 | 12,0 | 40,0 | 78,0 | 83,0 | 39,0 | 60,0 | 54,0 |
| SD | 3,8 | 3,3 | 5,0 | 2,0 | 4,0 | 3,3 | 8,6 | 5,2 | 6,8 | 3,8 | 3,3 | 6,9 |
| Improvement | - | - | - | - | - | - | 27,0 | 50,0 | 48,0 | 30,0 | 46,0 | 42,0 |

**Table S5.** Germination percentage of the pH, salinity, and iron experiment with 15 different combined treatments (RUN) for three different factors following a Box-Behnken Design (BBD). M1: Sebkhet Cherita; M2: Agareb coast; M3: Kraten. R1, R2, R3 and R4 (four replicates of 25 seeds each one). SD: standard deviation. Raw data.

| **REPLICATES** | **RUN** | **M1** | **M2** | **M3** |
| --- | --- | --- | --- | --- |
| R1 | 1 | 4 | 40 | 52 |
| R2 | 1 | 8 | 28 | 40 |
| R3 | 1 | 8 | 36 | 56 |
| R4 | 1 | 12 | 40 | 40 |
| R1 | 2 | 4 | 20 | 40 |
| R2 | 2 | 8 | 12 | 36 |
| R3 | 2 | 0 | 28 | 16 |
| R4 | 2 | 4 | 16 | 40 |
| R1 | 3 | 8 | 40 | 40 |
| R2 | 3 | 0 | 32 | 48 |
| R3 | 3 | 4 | 48 | 40 |
| R4 | 3 | 12 | 36 | 48 |
| R1 | 4 | 0 | 32 | 8 |
| R2 | 4 | 0 | 48 | 20 |
| R3 | 4 | 4 | 28 | 20 |
| R4 | 4 | 4 | 28 | 28 |
| R1 | 5 | 12 | 32 | 40 |
| R2 | 5 | 8 | 32 | 32 |
| R3 | 5 | 8 | 20 | 40 |
| R4 | 5 | 8 | 28 | 28 |
| R1 | 6 | 8 | 40 | 60 |
| R2 | 6 | 12 | 28 | 48 |
| R3 | 6 | 4 | 36 | 48 |
| R4 | 6 | 12 | 36 | 52 |
| R1 | 7 | 12 | 40 | 40 |
| R2 | 7 | 4 | 48 | 52 |
| R3 | 7 | 4 | 52 | 40 |
| R4 | 7 | 4 | 52 | 48 |
| R1 | 8 | 12 | 52 | 60 |
| R2 | 8 | 8 | 60 | 56 |
| R3 | 8 | 20 | 52 | 60 |
| R4 | 8 | 20 | 60 | 60 |
| R1 | 9 | 12 | 40 | 40 |
| R2 | 9 | 4 | 48 | 32 |
| R3 | 9 | 8 | 40 | 32 |
| R4 | 9 | 4 | 32 | 32 |
| R1 | 10 | 4 | 28 | 36 |
| R2 | 10 | 0 | 36 | 40 |
| R3 | 10 | 4 | 32 | 40 |
| R4 | 10 | 8 | 40 | 40 |
| R1 | 11 | 12 | 48 | 40 |
| R2 | 11 | 4 | 48 | 56 |
| R3 | 11 | 10 | 52 | 40 |
| R4 | 11 | 8 | 40 | 48 |
| R1 | 12 | 4 | 20 | 28 |
| R2 | 12 | 8 | 20 | 20 |
| R3 | 12 | 12 | 28 | 28 |
| R4 | 12 | 12 | 20 | 32 |
| R1 | 13 | 0 | 40 | 20 |
| R2 | 13 | 8 | 20 | 36 |
| R3 | 13 | 4 | 28 | 24 |
| R4 | 13 | 8 | 20 | 40 |
| R1 | 14 | 4 | 20 | 20 |
| R2 | 14 | 4 | 36 | 44 |
| R3 | 14 | 0 | 20 | 12 |
| R4 | 14 | 0 | 32 | 36 |
| R1 | 15 | 4 | 40 | 40 |
| R2 | 15 | 20 | 28 | 36 |
| R3 | 15 | 8 | 28 | 40 |
| R4 | 15 | 12 | 28 | 48 |
